# Remarks on Pathology of the Gums

**Published:** 1868-08

**Authors:** Harrison Ross

**Affiliations:** Quebec, P. Q.


### *Remarks on Pathology of the Gums.*

By Harrison Ross, D. D. S., Quebec, P. Q.

Although, strictly speaking, the dentist is limited in his treatment of inflammation or other pathological conditions of the gums, to the removal of causes which act mechanically as irritants and to topical applications, such as the various astringent, sedative and cauterizing agents, &c., &c., I think it highly important that we should all understand thoroughly, the various diseases peculiar to the gums; as a great deal of trouble and suffering may be saved by timely attention.

Should the case require surgical or medical treatment' the properly educated dentist will readily form a correct diagnosis, and refer the individual to his medical adviser, for proper treatment.

By possessing the requisite ability to discern cases of this kind, and placing them in the proper hands, the dental surgeon will benefit his own reputation and that of his profession.

There is a species of inflammation of the gums which, in some individuals, is so persistent, that it may with propriety be termed chronic inflammation. I have come across a few of these cases; the entire gum, but more particularly the inferior, becomes implicated in this variety of inflammation.

Among other symptoms of chronic inflammation, we find presented a thickened, soft, and very vascular appearance, with a tendency to grow over the teeth, and where there are broken down roots in the mouth, the gum is apt to envelope them entirely.

Dyspeptics, and persons suffering from gastric, intestinal, and hepatic inactivity, are, I think, more than others subject to this kind of sore gum. In many of these cases, the soreness of the mouth and gum being nearly sympathetic, the treatment indicated is first to get rid of the exciting cause by proper medication; after which, there will generally be but little difficulty in restoring the mouth to a healthy state.

A short time since, I had an opportunity of examining the mouth of a young lady who was at the time suffering from a peculiar form of *relitis*; the gums of this patient presented a very singular appearance, being considerably tumefied, the free edges moveable, so that an instrument could be passed a long way down between the gum and roots of all the teeth. There was so large a secretion of *epithelium* as to form a white coat over the entire body of the gums. This was thickest on the least elevated parts; easily rubbed off, and when removed, showed the *papillæ* very much more prominent than is usual. On pressure being applied to the edges of the gums, there was a slight discharge of fœtid, purulent matter. The most curious feature of this case, however, is, that the condition that I have described lasts but a few days at a time. It occurs about two or three times in the course of a year; and has continued to do so for about three years.

During the attacks, the pain and soreness which accompany the other symptoms, render the mastication of solid food a very difficult matter. Several physicians and dentists have examined this mouth, and attempted to effect a cure, but without success.

This young lady enjoys excellent health, her teeth are tolerably good, and free from deposits of tartar or other irritating causes.

It is my intention to watch this curious case, and I shall endeavor by proper treatmen to check its progress, when next the uneasiness about the teeth and gums, which precede its appearance, indicates that *relitis* is again about to set in.—*Canada Journal of Dental Science.*

